# Christmas

**Published:** 1873-12

**Authors:** 


HALL'S

### JOURNAL OF HEALTH.

VOL. XX.—DECEMBER, 1873.—NO. XII.

### CHRISTMAS.

"HERE comes old Father Christmas,
Welcome or welcome not;
I hope old Father Christmas
Will never be forgot."

☞ THIS December number will "come out" a month before Christmas, and, in order to have as much fun as possible in that joyous time, it would be well in the first place to arrange that there should be no debts coming due from you to others within two weeks before and two weeks after the holidays. It is not possible for any father or mother to be easy in their minds on Christmas Eve, while preparing to fill the stockings of the dear little ones, to be opened the next morning with such rapturous expectancy, to know that the day after a "heavy note" is due in the bank, or that a friend to whom you owe the money is looking to you with the utmost confidence that you will pay up, to enable him to meet his own maturing obligation. It is true that, by some fatality, the "first day of January" is made pay day by multitudes, but it ought not to be so, for the reasons named. If, by any mishap, or from want of forethought, you are already committed to the payment of money about the holidays, try and arrange, even if by a little sacrifice, to anticipate the payment a week or two, so as to have your mind clear of it, and that you may be able to enter into the enjoyments of the season with your little ones heart and soul, and have no cloud in your blue sky on that universally joyous occasion.

The "holidays" are a resting place for that vast number of persons who are always in debt; it is a kind of excuse that has more or less of foundation in it; nobody is expected to pay during that time, hence the easy promise is made, "I will attend to it after the holidays;" just like the struggling unfortunate who is somewhat relieved by being able to put a ticket on his door, "Closed by reason of death in the family," feeling assured that an importunate creditor will not intrude under the circumstances. And so it is "after the Fourth of July," "after Thanksgiving." What poor little mean equivocations does that remorseless monster, "debt," compel men to practice!

But do not be so selfish as to confine all your attention to securing your own enjoyment during the holidays. Can't you do a little for the comfort and happiness of others—for that poor struggling widow across the way, or that sick neighbor down the road? Go all through your house and hunt up the old clothing which you have cast off and do not expect to wear again. Get all the old shoes too much worn for your own use, such as have rips and holes; there are men who would very thankfully receive them.

"What are you going to do with all those old shoes?" I said, as I gazed at a whole pile of them one day in the magnificent parlor of Mrs. Dr. W.

"Oh, I'm sending them to the shoemaker to be mended."

"Why! are you and your daughters going to come out of your 'hundred foot front' into Fifth avenue in shoes with patches on them?"

"Oh no, doctor; there are some poor families on the east side of the town, and we are going to send them and some old clothing to them this cold winter weather; but we have all the patching done before we send them, because they might not have the money or the means to make them serviceable for some time to come, and they need them to-morrow."

What a thoughtful, loving heart beat in that woman's breast—worthy of a born "Knickerbocker," as she was. What refinement of delicacy and consideration! Shoddy or Corn Cob would have waited until some one called to beg their cast-offs, and then, with a scowl, would have thrown the old shoes on the floor, with a "clear out; don't bother me any more!"

But if Christmas is almost upon you before you chance to see this, and you have already parted with all your old clothing, do try and send a turkey or two, or a bushel of apples, or half barrel of flour, or keg of cranberries to the Five Points Mission House, to help spread a bounteous table to the poor little outcast children, who will never sit down to another good dinner until a whole year has passed away.

Maybe there is a sick mother near you—widowed, and poor, and sad; send her a full plate from your own Christmas dinner. And that young sewing girl, whom you have seen sitting at her window in the warm summer time, but has now "taken to her bed," and is slowly going down into the grave, send her a bunch of flowers, even if it costs but ten cents, for it is the last she will ever see. Before the early spring time, even long before the first daisy peeps from under the snow, she will have been mouldering in the grave; but anyhow that flower will gladden her sinking heart, and another thing that will gladden it more, that some one above her in social position had thought kindly of her.

Then there is your minister; surely you won't forget him. You can't forget him—the man whom you first send for when terrible sorrow comes into your dwelling. Think of him, not as a pauper object of charity, but as of a man to whom you and your country, and the whole world owe a debt which never can be paid—for without the influence, the work and the labors of the Christain minister, the globe would be a pandemonium of misrule, and sin, and cursedness and crime. Make him some gift, not for its value but for the countenance it gives, for the proof it is of your consideration, like the offering which the wise men of the east cast at the feet of the infant in the stable, glad to have the privilege of doing it. Well, then, send your minister a book, or some useful thing, whether "gold, or frankincense or myrrh," or a year's subscription to some religious newspaper, or literary magazine, or theological review, feeling all the while that, whether to your sick neighbor, or your clergyman, or the dying sewing girl, or the waifs at the Five Points, they are all God's children—they are all the purchase of a Saviour's blood, and that that same "Son of Man" is looking down upon you from his throne in immensity, saying, "Inasmuch as you have done it unto one of the least of these, my brethren, ye have done it unto me." The sweet "soft voice" may only be heard in your heart to-day, but it will be repeated in the hearing of angels and of men in that day for which all other days were made, when the angel, standing with one foot on the land and the other on the sea, shall proclaim that "time shall be no longer," and they who have done righteously shall go up with their Lord in the air.

Finally, reader, it may be to you the last chance you will ever have of doing this on a Christmas Day, for certain it is that some of you will never see another.

